# Awareness of euglycaemic diabetic ketoacidosis during pregnancy prevents recurrence of devastating outcomes: a case report of two pregnancies in one patient

**DOI:** 10.1186/s12884-021-04035-6

**Published:** 2021-08-12

**Authors:** Susanne Dargel, Ekkehard Schleußner, Christof Kloos, Tanja Groten, Friederike Weschenfelder

**Affiliations:** 1 Department of Obstetrics, University Hospital Jena, Am Klinikum 1, 07747 Jena, Germany; 2 Department Internal Medicine III, FB Endocrinology and Metabolic Diseases, Hospital Jena, Jena, Germany

**Keywords:** Preexisting diabetes, Euglycaemic ketoacidosis, Pregnancy, Stillbirth, Case report

## Abstract

**Background:**

Euglycaemic diabetic ketoacidosis (DKA) during pregnancy is a life-threatening obstetric emergency. It requires early identification and prompt action. Obstetricians’ knowledge about symptoms, diagnostic pitfalls and management during pregnancy and delivery need to be improved. We report a case of a young diabetic woman developing severe euglycaemic DKA in two consecutive pregnancies; the first pregnancy resulted in the most deviating outcome (i.e., intrauterine death), while the second pregnancy resulted in the delivery of a healthy newborn. Thus, the novelty of the case presented here is the possibility to demonstrate how the management of DKA in pregnancy can dramatically change outcomes.

**Case presentation:**

We report a case of a young diabetic woman in whom DKA was concealed by hyperemesis and oesophageal reflux. This woman presented to our delivery unit with severe euglycaemic DKA during her first pregnancy. While the mother’s condition could be successfully stabilized, the foetus died shortly after admission. Two years later, the same woman presented with similar problems. Repeated episodes of mild euglycaemic DKA could be successfully managed with consequent interdisciplinary treatment and close observation, leading to a good pregnancy outcome, i.e., the birth of a healthy child.

**Conclusion:**

Awareness of euglycaemic DKA needs to be increased to reduce the risk of severe complications during pregnancies in diabetic women. This case report demonstrates that increased awareness of DKA with immediate recognition and a successful multidisciplinary approach are mandatory for an positive pregnancy outcomes.

## Background

Diabetic ketoacidosis (DKA) is a rare but major risk during pregnancy among women with diabetes and endangers the life of the mother and foetus. Women with preexisting diabetes, especially women with type 1 diabetes, require insulin to maintain normal blood glucose and to inhibit lipolysis. The absence of insulin can therefore lead to hyperglycaemia and acidosis due to the lack of inhibition of lipolysis. DKA is an emergency and needs to be treated immediately. Euglycaemic DKA is even more dangerous because it is usually identified too late, since high blood glucose levels are absent in these cases. DKA occurs in 0.5–3% of all diabetic pregnancies (pregestational and gestational diabetes), and up to 30% of the cases of DKA during pregnancy are euglycaemic [1]. Triggers for DKA are continuous vomiting, prolonged hypocaloric intake or even starvation, acute infections, poor control of blood sugar or poor compliance with treatment, insufficient self-management of diabetes therapy, glucocorticoid therapy, ß-mimetics and stress or labour. In the absence of oral intake under the mentioned conditions, blood glucose can become low, here resulting in the disastrous decision to stop exogenous insulin by uninformed patients or health practitioners. Pregnancy itself can additionally trigger DKA due to the amount of contrainsulinogenic hormones such as human placental lactogen in combination with the reduction of buffering capacity because of pregnancy-induced respiratory alkalosis (compensatory renal loss of bicarbonate) and a significant rise in insulin demand during the second trimester. As a result, DKA in pregnancy often occurs even with low blood glucose levels. Foetal lethality depends on the severity of DKA and can reach up to 70% [1, 2].

Here, we present two consecutive pregnancies in one patient suffering from euglycaemic DKA in both pregnancies. The first resulted in intrauterine death of the child during a ketoacidotic crisis, while the second resulted in live birth of a late preterm but healthy baby girl. The aim of this case report is to raise awareness and to present treatment and management for this real and life-threatening complication in type 1 diabetes during pregnancy. Awareness and knowledge of management among obstetricians are mandatory to avoid fatal courses of DKA during pregnancy. The unique incidence of ketoacidosis during two consecutive pregnancies in one patient underlines the exceptionality of the case presented here.

## Case presentation

### Presentation of the 1st pregnancy

A 23-year-old Caucasian gravida 2 para 0 (spontaneous abortion at 6 weeks of gestation one year before) with type 1 diabetes (first diagnosis 8 years ago, HbA1c 6% before pregnancy) on an intensified insulin regimen (basal NPH insulin: 16 IE- 0-8 IE and prandial injections using insulin lispro on an insulin-to-carb ratio of 1:1) presented with symptoms of nausea and vomiting in her first trimester. She underwent standard hyperemesis therapy with administration of intravenous fluids, vitamin B complex and antiemetics several times in different hospitals and antiemetic suppositories during outpatient treatment. At 23 weeks of gestation, she presented again with persistent severe vomiting and abdominal pain, severe ketonuria and glucosuria in a euglycaemic situation (blood glucose 8–9 mmol/l). Because of haematemesis, gastroscopy was performed, and reflux oesophagitis with an axial sliding hernia not requiring intervention was diagnosed. Gastroenterologists recommended high-dose proton pump inhibitors (PPI) only. In addition to adaptation of the insulin regimen, hyperemesis treatment was accelerated, and therapy with PPI was started with 40 mg pantoprazole twice a day, resulting in the improvement of nausea and the elimination of ketonuria. At discharge, she had recovered and was in good condition. Nevertheless, one week later, further hospitalization became necessary because of another episode of impressive haematemesis (but without relevant impact on Hb levels), abdominal pain, ketonuria and glucosuria. Antiemetic and infusion therapy were extended with only moderate effects. Ketonuria persisted, while blood sugar values remained within the target range. During the hospital stay, a third-trimester scan was performed and revealed a eutrophic foetus with normal sonoanatomy and echocardiography. Four days later, when abdominal pain and vomiting calmed, the patient discharged herself against medical advice, although ketonuria still persisted.

Three weeks later, at 27 weeks of gestation, the woman was transferred to our delivery room from a peripheral hospital due to persisting severe haematemesis. At arrival, she was in deteriorated general condition. Foetal status was checked immediately by ultrasound since no proper cardiotocography could be performed by the midwives upon admission. Foetal ultrasound revealed a foetal heart rate of approximately 110 bpm with an atypical arrhythmic pattern of the foetal heart, which was probably a sign of cardiac decompensation with lethal arrhythmias. Severe ketonuria and glucosuria were diagnosed when blood glucose was 8 mmol/l. At the referring hospital, which lacked an obstetric department, no cardiotocographic control of the foetus was performed. Furthermore, insulin delivery was stopped by the health care providers since the patient was starving for 24 h and oral food intake was not possible. We immediately started intravenous insulin therapy along with intravenous rehydration with normal saline. Approximately 30 min after initiating therapy, foetal monitoring revealed critical bradycardia of the foetus, which was confirmed by ultrasound. In that critical, unstable maternal metabolic condition, we decided against emergency c-section, and foetal demise in the course of euglycaemic diabetic ketoacidosis was diagnosed within a couple of minutes. Two hours after starting the therapy, the BGA still showed severe metabolic acidosis with signs of respiratory compensation (pH 7.25, BE − 16 mmol/l, HCO3–9.6 mmol/l, Lac 1.9 mmol/l, pCO2 2.97 kPa; Glc 13.2 mmol/l), thus confirming the diagnosis of severe euglycaemic DKA. After DKA had resolved, emergency gastroscopy was performed, PPI therapy was extended, and the induction of labour was started with misoprostol. Twenty-four hours later, the patient gave birth to a stillborn female (birth weight 1040 g; 68. percentile). She was dismissed two days later in a balanced metabolic condition.

### Presentation of the 2nd pregnancy

Three years later, the same woman presented with her second pregnancy at the 15th week of gestation, again due to vomiting in acidotic condition. At that time, HbA1c was 7.2% under the intensified insulin regimen (insulin detemir 13 IE-0-10 IE and prandial injections using insulin lispro and an insulin-to-carb ratio of 1:1). With the negative outcome of the last pregnancy in mind, we were prepared to react immediately. An interdisciplinary team of obstetricians and diabetes specialists was put together to plan and implement the optimal management of the pregnancy. Management could be realized in an outpatient setting and pregnancy advanced.

**Fig. 1:**
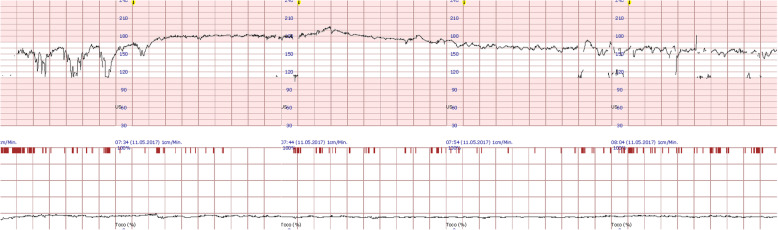
Foetal monitoring in the 2nd pregnancy during diabetic ketoacidosis (DKA) at 27 weeks of gestation. Typical but not specific changes associated with DKA in cardiotocography (CTG) are loss of variation of foetal baseline and variable decelerations, which can be seen in the first 30 min of this CTG. The effects of DKA therapy (here: saline liquids and adapted insulin therapy) can be observed within 30 min as the foetal heart rate and CTG pattern normalize (decelerations disappear, heart rate slows down to 160 bpm, and variability reappears)

After 27 weeks, the patient was admitted due to diabetic ketoacidosis. The blood gas analysis revealed a pH of 7.28, BE − 12.5 mmol/l, HCO3–13.0 mmol/l, pCO2 3.68 kPa, Lac 1.2 mmol/l. Concomitantly, foetal monitoring showed cardiotocography with the typical pattern for maternal ketoacidosis with loss of variation of foetal baseline and variable decelerations (Fig. 1). The observed changes in the cardiotocography pattern immediately resolved upon treatment with ketoacidosis. To closely monitor the mother and foetus and avoid repeated critical metabolic situations, we decided to keep the patient hospitalized until delivery. Nevertheless, repetitive episodes of incipient metabolic derailment accompanied by vomiting occurred and could all be treated immediately and successfully. A psychological cotreatment was organized to explore whether self-induced vomiting or self-induced hypoglycaemia by overdosing insulin injections could be the reason for these repetitive clinical patterns, such an inpatient situation. However, the pregnant woman was not willing to cooperate, and this assumption could never be confirmed or excluded. Finally, we decided to have insulin injections exclusively executed by medical professionals, and the metabolic situation remained stable until 36 weeks of gestation.

At 36 weeks of gestation, we made an interdisciplinary decision to terminate the pregnancy because the patient was emotionally very distressed due to her history, and we feared another metabolic derangement. Labour was induced with prostaglandins in a stable glycaemic situation, and the patient gave birth to a healthy girl (weight: 2840 g, 48. percentile; height 48 cm, APGAR 7/8/9 pHa 7.09 and pHv 7.19). Because of mild respiratory problems and an initial blood sugar of 1.8 mmol/l, the girl was admitted to our neonatal intensive care unit for one night. The next day, the mother and daughter were happily reunited. Blood sugar levels of the mother remained stable under the intensified insulin regimen (insulin detemir 5–0-0-8 IE and insulin lispro according to an insulin-to-carb-ratio 0,5:1). Lactation started on time. The mother and child were discharged together five days after delivery, and both of them were in good and healthy condition.

## Discussion and conclusion

Hyperglycaemic DKA is a well-known complication in diabetic patients, especially those with insulin-dependent diabetes mellitus. Euglycaemic DKA is rare, has an insidious onset and is still relatively understudied. During pregnancy, long-lasting ketoacidosis can cause neurological complications and can end up in foetal death [3, 4]. Therefore, it constitutes an obstetric emergency and requires early identification and treatment. Euglycaemic DKA is characterized by ketoacidosis with electrolyte derangement and only marginal or absent elevation of serum glucose. Due to the higher levels of hormones with contrainsulin effects and the consecutive strong increase in insulin demand in the second and third trimesters combined with emptied glycogen stores due to pregnancy-related metabolic changes, the risk for euglycaemic DKA increases during the second half of pregnancy. In addition, pregnancy-induced respiratory alkalosis supports the development of DKA at lower glycaemic levels [1, 3]. Additional risk factors may further escalate pathophysiology. In our case, persistent vomiting with haematemesis, caused by reflux oesophagitis with an axial sliding hernia, supported the rapid development of euglycaemic DKA. Abdominal pain and vomiting are typical signs of DKA. Unfortunately, in our case, these classical symptoms of ketoacidosis were concealed by persistent hyperemesis in early pregnancy and later on by the diagnosis of oesophageal reflux disease during the first pregnancy.

DKA during pregnancy is associated with various foetal immediate and long-term complications [5]. Although literature dealing with long-term outcomes is rare, impairment of brain development (lower IQ) and the development of autistic children have been described [6, 7]. Immediate foetal complications include high foetal mortality rates of 27–35%. Several reasons for foetal lethality have been discussed in previous studies. DKA-associated hypovolemia causes decreased uteroplacental blood flow and increased concentrations of catecholamines, leading to foetal hypoxemia. Transfusion acidosis with electrolyte dysbalance and hyperlactacetemia worsens foetal hypoxemia. Additionally, lethal cardiac arrhythmias, as seen in the first pregnancy accompanied by foetal demise, might occur due to foetal hyperinsulinaemia-induced hypokalaemia [1, 2, 8]. During the 2nd pregnancy described in our report, we observed typical DKA-related changes in the CTG (Fig. 1), which disappeared after correcting the DKA. It should be noted that acute severe DKA should never result in immediate emergency delivery, as this would harm the mother and most likely not save the child. If delivery is inevitable, as in cases where maternal condition worsens despite aggressive therapy, it is associated with high maternal morbidity and mortality [1, 5, 9].

Prevention of (euglycaemic) DKA should be a key aspect in educating and managing pregnant diabetic women. They have to receive adequate preconceptional counselling as well as education about precipitating factors, signs and symptoms of diabetic ketoacidosis. Instructions about the importance of compliance with diet, prenatal visits, measurement and recordings of glucose values are necessary, as well as compliance with therapy. It must be emphasized that insulin is necessary at all times for women with type 1 diabetes. In episodes of low blood glucose levels and oral intake of calories, immediate presentation to the next hospital is not immediately necessary. Self-control of urine-ketone bodies must be integrated into the daily self-care of every pregnant diabetic woman. Furthermore, all obstetricians and medical professionals involved in the emergency service have to be aware of the possibility of DKA in pregnancy and its jeopardizing consequences for mother and foetus. Insulin treatment should never be stopped.

Our cases represent the two contrary outcomes of euglycaemic DKA in two consecutive pregnancies of the same patient. Thus, the strength of the presented case report(s) is to demonstrate the immediate impact of treatment and management of DKA during pregnancy in one patient under exclusion of patient-to-patient variances. The first case presents a pregnancy with a poor obstetric outcome due to several unfavourable circumstances, such as typical symptoms of DKA being concealed by corollary problems and inexplicable mistreatment by stopping insulin treatment in a type 1 patient. The 2nd case emphasizes the positive effect of interdisciplinary individualized management leading to the desired delivery of a healthy child. Thus, this case report clearly demonstrates the effects of appropriate treatment. Remarkably, the patient specifically sought our help and treatment during her second pregnancy and even consented to permanent inpatient admission for the remaining 10 weeks of her pregnancy to avoid a repeat of the fatal outcome. Despite this willingness to place herself in our care, we, as a team of therapists, were not able to completely prevent ketoacidotic episodes or understand their origin.

Euglycaemic DKA is a rare but serious and highly challenging complication of diabetes in pregnancy that jeopardizes the mother and the foetus. The prompt recognition of triggering factors, adequate rehydration, proper insulin administration and correction of electrolyte imbalance are the key points in the treatment. Knowledge and training of obstetricians, diabetologists and pregnant diabetic patients as well as multidisciplinary treatment approaches combined with continuous maternal and foetal monitoring are mandatory to improve the overall morbidity and mortality.

## Data Availability

Please contact the corresponding author at tanja.groten@med.uni-jena.de for data requests.

## Abbreviations

DKA: diabetic ketoacidosis
CTG: cardiotocography
